# Communicating Nutritional Knowledge to the Chinese Public: Examining Predictive Factors of User Engagement on TikTok in China

**DOI:** 10.3390/bs14030201

**Published:** 2024-03-02

**Authors:** Min Zhu, ShaoPeng Che

**Affiliations:** School of Journalism and Communication, Tsinghua University, Beijing 100084, China; zhum21@mails.tsinghua.edu.cn

**Keywords:** nutrition communication, science popularization, user engagement, communication strategy, TikTok, Douyin

## Abstract

Objective: This study aims to identify content variables that theoretical research suggests should be considered as strategic approaches to facilitate science communication with the public and to assess their practical effects on user engagement metrics. Methods: Data were collected from the official Chinese TikTok account (Douyin) of the Nutrition Research Institute of China National Cereals, Oils and Foodstuffs Corporation, China’s largest state-owned food processing conglomerate. Dependent variables included likes, shares, comments, subscription increases. Independent variables encompassed explanation of jargon (metaphor, personification, science visualization), communication remarks (conclusion presence, recommendation presence), and content themes. Descriptive analysis and negative binomial regression were employed, with statistical significance set at 0.05. Results: First, subscription increases were positively associated with personification (*p* < 0.05, 0.024) and science visualization (*p* < 0.01, 0.000). Second, a positive relationship existed between comments and communicator recommendations (*p* < 0.01, 0.000), while presenting conclusions negatively correlated with shares (*p* < 0.05, 0.012). Conclusions: Different strategies yielded improvements in various engagement metrics. This can provide practical guidance for communicators, emphasizing the need to balance scholarly presentation with sustaining appealing statistics.

## 1. Introduction

Public nutrition education in China represents a unique paradigm within the Global South, characterized by a centralized model for operationalizing public health initiatives [1]. The strategic emphasis on new media, particularly social media, has become pivotal following the central government’s commitment to enhancing nutritional literacy [2,3]. Amid the COVID-19 pandemic [4], TikTok has emerged as a prominent domain, boasting over 750 million daily active users with an average viewing duration of 2.5 h [5,6]. Within this digital landscape, nutrition communication is a leading video category with daily health popularization content attracting more than 200 million users [7]. Notably, content addressing low-sodium, low-sugar, and low-fat dietary practices has witnessed substantial annual increases in viewing numbers [8], reflecting a growing public demand for professional nutritional knowledge [9].

In response to the vital role assumed by nutrition professionals in ensuring the daily health care of the public, dietitians have initiated measures to disseminate information aimed at reaching and educating users. However, they face challenges conveying technical jargon to the public, expressing reservations about simplifying scientific concepts on TikTok [10]. Collaborative efforts between nutrition and communication experts have aimed to provide guidance, ensuring accurate information dissemination and comprehension among a broader audience. Strategies include abandoning insignificant terminology, contextualizing scientific data within individuals’ daily lives, specifying recommended intake frequency, and identifying target demographics [11]. Several studies have highlighted the effectiveness of figurative speech in simplifying technical terms by altering the relationships between designated concepts [12,13]. Additionally, the integration of science visualization has proven instrumental in enhancing audience perception by offering tangible interpretation of abstract notions [14,15], resulting in heightened engagement compared to traditional text formats [16]. 

Concerns about the misrepresentation of scientific findings are legitimate, yet it is crucial to recognize that scientists may inadvertently overlook the communication of scientific summaries and advice. This oversight often stems from their familiarity with the information [17], and instances of misrepresentations are comparatively rare, given the scrutiny of the scientific community and vigilant public oversight. Moreover, research findings underscore that content possessing practical utility and the potential for seamless integration into everyday life tends to garner heightened popularity [18]. This highlights the importance of addressing concerns about misrepresentation and emphasizing the practical relevance of scientific information for wider user engagement.

The existing literature extensively examines the favorable effects of social media on behavior intervention. In a controlled study targeting adult women with suboptimal fruit and vegetable consumption, exposure to healthy eating blogs demonstrated significant improvements in intervention outcomes. Specifically, the exposed group exhibited elevated diet knowledge, enhanced attitude, increased self-efficacy, and greater motivation to enact change. As a prevalent form of content on social media, particularly notable for their knowledge-translation capabilities, convenience and interactive features, blogs have proven instrumental in effecting positive lifestyle behavior changes [19]. This illustrative case aligns with a broader body of research [20], indicating that social media contributes to more successful intervention outcomes by fostering social norms and empowering individuals to embark on health improvements [21]. 

Recent scholarly inquiries underscore the critical role of user engagement metrics as an influencing factor of health intervention outcomes, encompassing number of likes, shares, comments, and subscriptions per video. The act of clicking ‘like’ as an instant impression imposes no effort burden on users; however, it serves as a key engagement metric indicating user interest, whereas sharing not only signifies viral reach [22] but also has the potential to influence those in the users’ social circle. Comments and subscriptions constitute active forms of engagement, with the former serving as a platform for active and public deliberation and the latter indicative of the audience’s long-term commitment [23]. Current research emphasizes discerning shared attributes among highly engaged posts [24], investigating the impact of stylistic and informational elements on popularity [25], and exploring the strategies employed by nutrition professionals on social media platforms [26]. 

While some attention has been given to content-based variables and their impact on user engagements, few studies have delved into the realm of nutritional knowledge dissemination and its effect evaluation. The COFCO (China National Cereals, Oils and Foodstuffs Corporation) Nutrition Research Institute has emerged as a noteworthy research target. As the inaugural enterprise research and development center dedicated to investigating the Chinese population’s nutritional needs and metabolic mechanisms, its parent company consistently ranks among the Fortune Global 500 companies [27]. The research institute, a pioneer in establishing a public account for disseminating nutrition knowledge, has garnered accolades, such as the Outstanding Group for Science Popularization, awarded by the Ministry of Science and Technology of China [28]. These distinctions, reflective of exemplary achievements, set benchmarks for other enterprises to emulate and align with national initiatives. Analyzing how the COFCO Research Institute communicates with the public on TikTok provides valuable insights into the evolving landscape of digital health and nutrition education in China. 

**Figure 1:**
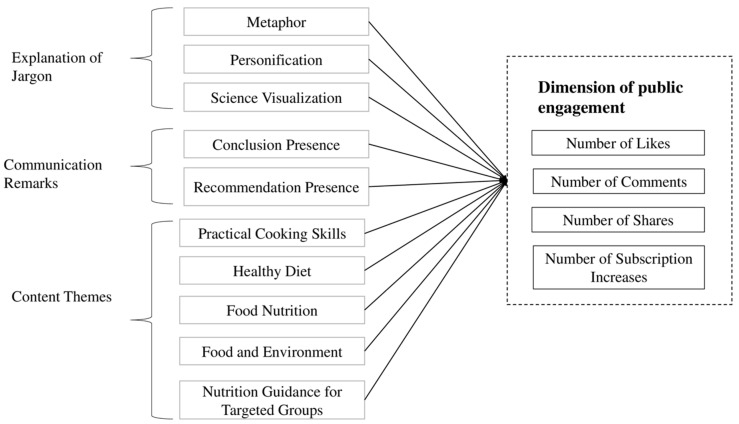
Research model for evaluating the impact of nutrition knowledge dissemination on TikTok.

This study endeavored to bridge existing gaps in the literature by addressing the following research questions. First, an exploration was conducted into the impact of employing metaphors, personification, and scientific visualization on the user engagement metrics of the COFCO Nutrition Research Institute. Second, an investigation delved into the influence of communication remarks, specifically presenting definitive scientific conclusions and offering recommendations, on user engagement metrics. Third, a detailed analysis was undertaken to discern how content themes contribute to variations in user engagement metrics. The conceptual framework illustrating these research inquiries is depicted in Figure 1.

## 2. Materials and Methods

### 2.1. Account Selection

This study employed both descriptive and regression analyses, utilizing data extracted from the TikTok account of the COFCO Nutrition Research Institute. The selection of this particular account was deliberate and informed by several key considerations. First, the account stands out as one of the rapidly expanding official research channels dedicated to public nutrition popularization. Since its inception on 24 July 2022, within a year of operation, the account has disseminated 180 videos, accumulating over 4.5 million likes and amassing a follower base exceeding 150,000.

Second, the account’s objectives and published content align closely with promoting nutrition and health information pertinent to individuals’ daily lives. Notably, the selected account distinguishes itself by adhering to a consistent format, featuring a professional expert delivering monologues against a laboratory backdrop. This uniformity, with videos consistently ranging from 30 to 40 s, positions the COFCO Nutrition Research Institute as an optimal target for assessing the research questions posed in this study.

### 2.2. Data Collection

Data collection encompassed all content posted by the COFCO Nutrition Research Institute from 1 September 2022 to 30 June 2023, resulting in the acquisition of 126 videos. Following communication with the COFCO Research Institute, videos produced by a third-party Multi-Channel Network service provider were meticulously excluded. Subsequently, 81 videos, exclusively created and managed by the research institute, were retained for analysis.

The study aimed for data saturation, ensuring that the selected samples effectively represent and facilitate in-depth analysis of the research questions. Prioritizing the depth of analysis holds greater significance than pursuing a larger sample size, aligning with the study’s theoretical framework and objectives. Despite the limited sample size, the subsequent results section will demonstrate the study’s robust statistical power, offering meaningful findings.

Notably, this research pioneers the investigation of public digital nutrition education and its performance evaluation, with a strong emphasis on content-based variables. While the sample size is constrained, this pioneering approach lays a foundation for future explorations in this underexplored domain.

Access to data was authorized and facilitated through collaboration with the COFCO Nutrition Research Institute. The dataset comprised seven types of information: video title text, content of the video, posting time, number of likes, number of shares, number of comments, and number of subscription increases. Institutional Review Board approval was not required because of the nature of this study.

### 2.3. Operationalization of Variables 

User engagement on the official TikTok account of the COFCO Nutrition Research Institute was assessed across four dimensions—number of likes, shares, comments, and subscription increases—all meticulously recorded. 

Video title text was systematically collected by manually extracting complete titles and quantifying the word count using Excel. The posted time signifies the precise date and time of video upload to the TikTok platform for public viewing.

Jargon, defined here as using technical and disciplinary language rather than obscure or pretentious terms [29], was measured using metaphors, personification, and science visualization. The measure of metaphor involves explaining jargon through analogies to other things with similar characteristics, while personification entails attributing human attributes to illustrate terminology. The use of multiple metaphors in a video warranted a marking of 1, as does employing personification. Communication remarks were evaluated based on the presentation of definite scientific conclusions and offering recommendations, with more than one recommendation considered as 1. 

Content themes were categorized into five distinct types: practical cooking skills, healthy diet, food nutrition, food and environment, and nutrition guidance for targeted groups. Two authors conducted a comprehensive review and inductively coded the videos to identify key themes. Each video was assigned a single theme code according to its primary focus. In cases where multiple themes seemed applicable, the authors deliberated until reaching a consensus on the primary focus. Practical cooking skills encompassed videos featuring cooking recipes, techniques, and ingredient preparation. Videos categorized under healthy diet provided information on fostering healthier eating habits. In contrast, food nutrition videos centered on abstract information regarding the benefits and risks of nutrients to the human body. Food and environment addressed food production, safety, responsible consumption, and food sustainability concerns. Nutrition guidance for targeted groups involved videos tailored to specific dietary needs, such as for the elderly or individuals with high blood pressure, aimed at promoting their health and well-being. See Table 1 for examples.

### 2.4. Coding Method

This study employed an iterative, open coding process, initially forming themes based on references and the latest research in nutrition science and public communication. The codebook underwent refinement and finalization through repeated coding checks and comparison with theoretical memos. Practically, content themes were identified by analyzing video titles and content. When a video encompassed more than one theme, the decision was guided by hashtags in the video title, emphasizing the central theme presented in the initial 10 s of the content. Communication remarks and the explanation of jargon were discerned through the identification of relevant specific phrases or images within the videos.

#### Coders and Reliability Test

Given the nutrition communication focus of the video content, two graduate students specializing in biology and communication studies were engaged in the coding process to ensure elevated coding validity. Before formal coding, 20% of the samples were randomly selected for initial coding without allowing communication between coders. The formal coding phase commenced only when the κ values reached 0.9 or higher, ensuring a consistent understanding of coding standards among the coders.

In cases where reliability thresholds were not met, additional training and codebook clarification were undertaken, specifically emphasizing contentious categories. Reliability was ultimately achieved through three rounds of meticulous training. The confidence coefficients derived from this reliability assurance process are presented in Table 1.

### 2.5. Statistical Analysis

All data were entered and analyzed using SPSS version 27.0 (SPSS Inc., Armonk, NY, USA, 2020). The study first obtained a frequency table of counts and percentages and then ran descriptive analyses and binominal regressions to investigate the distribution of content themes, engagement metrics, and the relationships between techniques and engagement metrics. Statistical significance for all tests was set at 0.05. 

## 3. Results

### 3.1. Descriptive Analysis

#### 3.1.1. Frequency Distribution of Content Themes

Table 2 comprehensively summarizes the content themes derived from the 81 validated samples. The analysis revealed that “Healthy Diet” emerged as the predominant theme, constituting 40.74% of the videos. Following closely were themes centered around “Food Nutrition” (17.28%) and “Food Environment” (16.05%). Additionally, 13.58% of the videos were dedicated to “Nutrition Guidance for Targeted Groups,” while “Practical Cooking Skills” accounted for 12.35%. 

#### 3.1.2. Descriptive Analysis of User Engagement

Table 3 provides a detailed descriptive statistical analysis of the user engagement metrics, revealing substantial variations in the collected data. Notably, the maximum values for likes, shares, comments, and subscription increases exhibit at least a hundredfold difference from the minimum values. The maximum values also surpass three standard deviations from the mean, underscoring the appropriateness of employing the median rather than the mean to portray overall performance accurately. 

The analysis of frequency tables for engagement metrics unveils that 32.10% of videos (*n* = 15) garnered fewer than 20 shares, whereas 3.7% of videos (*n* = 4) secured 500 or more shares. Similarly, 9.88% of videos (*n* = 9) amassed over 200 comments. The pattern persists in likes, where 30.86% of videos received fewer than 300 likes (*n* = 22), with 12.35% (*n* = 7) acquiring fewer than 100 likes. Furthermore, 20.99% of videos (*n* = 17) garnered five or fewer subscription increases.

The comprehensive analysis presented in Table 4 reveals distinctive patterns across various themes and user engagement metrics. When considering median statistics, “Practical Cooking Skills” emerges as the leading theme in terms of the number of likes (median = 1529.5, SD = 2508.037), shares (median = 80.5, SD = 603.248), and subscription increases (median = 81, SD = 120.626). Conversely, the “Food and Environment” theme takes precedence in the number of comments (median = 114, SD = 249.23).

In contrast, “Food Nutrition” ranks lowest in the number of likes (median = 469.5, SD = 958.839) and shares (median = 22, SD = 39.224). “Healthy Diet” occupies the lowest position in the number of comments (median = 34, SD = 126.811), while “Nutrition Guidance for Targeted Groups” exhibits the lowest number of subscription increases (median = 11, SD = 170.418). 

### 3.2. Negative Binomial Regression

#### 3.2.1. Research Question 1

RQ1 delved into the impact of metaphor, personification, and science visualization to elucidate scientific concepts on user engagement metrics. In Model 1 (Table 5), it is evident that metaphor usage exhibits a negative association with the number of subscription increases at the 0.01 significance level (regression coefficient = −0.974, z = −3.647, *p* = 0.000 < 0.01). This implies that a one-unit increase in metaphor usage corresponds to a 0.378-times decrease in subscription decreases, as indicates by the odds ratio. Conversely, personification (regression coefficient = 0.441, z = 2.262) and science visualization (regression coefficient = 0.478, z = 3.534) demonstrate significant positive impacts on the number of subscription increases at the 0.05 and 0.01 significance levels, respectively. The odd ratio values are 1.554 and 1.613, signifying that a one-unit increase in personification and science visualization leads to 1.554- and 1.613-times increases in subscriptions.

Negative binomial regressions were also conducted to explore associations between these variables and the number of likes, shares, and comments, revealing no significant associations.

#### 3.2.2. Research Question 2

RQ2 explored the influence of scientific conclusions and recommendations on user engagement metrics. In Model 2 (Table 5), presenting scientific conclusions exhibits a negative correlation with the number of shares (regression coefficient = −0.582, z = −2.511, *p* = 0.012 < 0.05). The odd ratio of 0.559 indicates that a one-unit increase in scientific conclusions corresponds to a 0.559-times share decrease. No significant relations are found between recommendations and the number of shares, signifying that suggestions did not impact the sharing count.

Regarding the number of comments (Model 3), offering recommendations displays a positive association, with statistical significance at the 0.01 level (regression coefficient = 0.924, z = 4.025, *p* = 0.000). The odd ratio of 2.518 suggests that a one-unit increase in recommendations leads to a 2.518-times increase in the number of comments. 

Notably, the study reveals associations of these variables with the number of shares and comments, while no significant relations are identified with the number of likes and subscription increases.

#### 3.2.3. Research Question 3

Table 6 presents the outcomes of negative binomial regression analysis, exploring the association between five content themes (practical cooking skills, healthy diet, food nutrition, food and environment, and nutrition guidance for targeted groups) and user engagement metrics (number of likes, shares, comments, and subscription increases). RQ3 investigated the relationship between content themes and these key user engagement metrics.

Table 6 focuses on subscription increases as the key metric. The regression analysis revealed that nutrition guidance for targeted groups and food nutrition did not significantly impact subscription increases (*p* > 0.05). However, practical cooking skills (regression coefficient = 2.010, z = 7.239, *p* = 0.000 < 0.01), healthy diet (regression coefficient = 0.772, z = 4.412, *p* = 0.000 < 0.01), and food and environment (regression coefficient = 1.395, z = 5.611, *p* = 0.000 < 0.01) demonstrated significance at the 0.01 level. Notably, an increase in practical cooking skills correlate with a 7.463-times rise in subscription increases, while food and environment and healthy diet themes led to 4.033- and 2.165-times increases, respectively. 

Regarding the number of comments, the regression model indicated a noteworthy positive correlation for four themes, excluding food nutrition. The regression coefficients were healthy diet (0.398, z = 2.267, *p* = 0.023 < 0.05), practical cooking skills (1.064, z = 3.824, *p* = 0.000 < 0.01), food and environment (1.311, z = 5.269, *p* = 0.000 < 0.01), and nutrition guidance for targeted groups (0.772, z = 2.888, *p* = 0.004 < 0.01), exhibiting significance at the 0.01 and 0.05 levels, respectively. The odd ratio indicated that the food and environment theme had the most substantial impact on the increase in comments, suggesting a 3.710-times greater increase when environmental and food content increased by one unit. 

Examining the association between content themes and the number of shares revealed statistical insignificance only for food nutrition (regression coefficient = −0.211, z = −0.907, *p* = 0.364 > 0.05). However, the other four themes showed significance at the 0.01 level: practical cooking skills (regression coefficient = 1.960, z = 7.051, *p* = 0.000 < 0.01), healthy diet (regression coefficient = 0.511, z = 2.909, *p* = 0.004 < 0.01), food and environment (regression coefficient = 0.782, z = 3.138, *p* = 0.002 < 0.01), and nutrition guidance for targeted groups (regression coefficient = 0.762, z = 2.848, *p* = 0.004 < 0.01). The odd ratio for practical cooking skills indicated the highest increase in sharing among the themes, with the number of 7.098. 

All five themes demonstrated statistical significance at the 0.01 level regarding the number of likes. Practical cooking skills had the highest odd ratio among the themes, with a value of 6.595. This implies that the change (increase) in likes was 6.595 times greater when practical cooking skills were increased by one unit.

## 4. Discussion

This study explored how nutritional scientists communicate with the public on TikTok and empirically examined its user engagement, and placed emphasis on how content-related variables that were theoretically posited can better help the public’s understanding of science. Few studies have organically integrated practical guidance for science communicators into examining their actual audience effects. More importantly, the rationale for this study was situated in how to have appealing user engagement metrics while also complying with the broader goal of the public communication of science. 

First, findings revealed that using personification and science visualization to explicate scientific jargon significantly correlated with increased account subscriptions. Conversely, adopting metaphors did not exhibit a positive association with user engagement metrics. This conclusion aligned with the insights offered by Borowiec et al.’s research [30], emphasizing the prerequisite for scientists to possess ample knowledge in replacing jargon with accessible terms before incorporating metaphors. 

In the second key finding, the study elucidated that nutrition communicators presenting definite scientific conclusions to the audience negatively impact the number of shares. In contrast, a positive correlation was observed between the provision of recommendations and the number of comments. The conveyance of definite conclusions imparts a sense of finality, leaving minimal room for personal interpretation or engagement. This trend underscores the intricate dynamics of online sharing behavior, where engagements often stem from the inclination to initiate discussions or provide fresh perspectives within an ongoing public discourse [31]. Conversely, nutritional communicators who furnish recommendations serve as a bridge between abstract concepts and practical applications. This connection establishes a sense of personal relevance among the audience, compelling them to share their thoughts, experiences, and inquiries through comments.

In the third pivotal observation, it is noted that the theme centered around food and environment elicited the highest number of comments, suggesting that comments are likely spurred by the novelty and curiosity inherent in the video’s theme. A specific instance in our sample, focusing on the correlation between cow flatulence, greenhouse gas emissions, and climate change, garnered many comments. The surprising revelations about the digestive process of cows, a major contributor to methane emissions, challenged conventional assumptions, prompting viewers to express their astonishment or seek further clarification. This phenomenon indicated the significant impact of counterintuitive information, fostering a willingness among viewers to engage through comments. Moreover, such content aligned with the entertainment-centric nature of social media platforms [32]. This outcome underscored the substantial potential for the in-depth exploration of this theme. By amalgamating the innate curiosity of the public with research areas of governmental and international concern, particularly in the realm of sustainable and healthy food creation, this theme possesses considerable prospects for further investigation.

In the concluding insight, the theme around food nutrition exhibited no statistically significant association with the number of shares, comments, and subscription increases yet displayed a robust correlation with the number of likes. This discovery aligned with Bhattacharya et al. [33], indicating that audiences are more inclined to appreciate positive and reassuring content. However, considering nutritional information is relatively well-established, providing more predictable content tends to evoke less curiosity, resulting in lower engagement across other metrics. Surprisingly, the study observed that the theme of nutrition guidance for targeted groups generated high engagement metrics without significantly impacting subscription increases. This discrepancy suggested that viewers, having already obtained the sought-after information, experienced diminished motivation to subscribe for additional content, as their immediate informational needs were adequately addressed.

## 5. Conclusions

This investigation underscored the theoretical viability and practical efficacy of employing techniques such as explaining jargon and communication remarks to enhance audience engagement in nutritional science communication. However, it is crucial to note that while these variables demonstrated statistically significant associations with specific engagement metrics, they were not uniform across all dimensions. 

A few limitations of the study should be addressed. Although the TikTok account of the COFCO Research Institute emerged as a suitable target for the current research, it should be noted that engagement metrics were influenced by subscriber demographics and target audience. Whether the results of this research are applicable to other nutrition communicators and in a global context is worthy of investigation [34,35]. Analyses grounded in cross-platform comparative studies between regions of the Global South and the Western Hemisphere, alongside the utilization of big data analysis, have the potential to yield more nuanced insights and enhance the broader applicability of the current study. In addition, a longer sampling time and larger dataset could yield more comprehensive results.

Given the nascent stage of nutritional communication and user engagement, ample room remains for further exploration. By establishing correlations between content variables and engagement metrics, this study lays the groundwork for future researchers to delve deeper into the operational dynamics of TikTok accounts. A qualitative approach in future research could unravel the intricacies of content selection processes, the strategic considerations guiding communicators’ choices, and their adherence to platform rules. By scrutinizing these factors, a more comprehensive understanding of the strategies employed by communicators to maximize engagement may emerge, contributing to a nuanced comprehension of the landscape of nutritional science communication on TikTok.

## Figures and Tables

**Table 1:** Operational definitions, examples and confidence coefficients to examine TikTok videos.

Variables	Operational Definition	Examples	Intercoder Reliability
**Explanation of Jargon**			1.00
Metaphor	Using parallel concepts to help illustrate science terminology	Lutein resides within the macula of the eye, serving as a shield that absorbs 40% to 90% of incoming blue light, similarly to equipping the eyes with a protective filter.	1.00
Personification	Applying human attributes to discuss a scientific term	L-arabinose can compete with sucrose in the intestines, taking the enzyme that digests sucrose away from sucrose and preventing this absorption trip.	1.00
Science Visualization	Usage of visual tools such as infographics and images to illustrate jargons	N/A	1.00
**Scientific Message Attributes**			1.00
Conclusion Presence	Science communicator gives definite and scientific conclusion	Natural vitamin E is better than synthetic vitamin E in every way.	1.00
Recommendation Presence	Science communicator offers tangible advice to the audience	The intake of natural vitamin E is also recommended in the daily diet.	1.00
**Content Themes**			1.00
Practical Cooking Skills	Giving recipes, cooking methods and culinary tips	How do you determine the temperature of the oil for stir-frying without a thermometer?	1.00
Healthy Diet	Providing knowledge about healthy eating habits, debunking myths, and encouraging diverse food intake	I’ve heard that the delicious taste you get from frying things in palm oil is something we trade for our health! Is this a rumor?	1.00
Food Nutrition	Educating the public about functions of different nutrients	The chemical structure of plant sterols and cholesterol is very similar, only because of the side chain structure of the C24 position on the extra group, the two on the human body’s role, is a world of difference.	1.00
Food and Environment	The impact of nutrients on the environment and the broader social context of sustainability eating	The Green Magic of Biofuels: Why Ethanol Reduces Carbon Emissions	1.00
Nutrition Guidance for Targeted groups	Addressing nutrition-related topics and giving tailored advice to a specific group	Scientists have discovered klotho, a protein that may be able to awaken memories which could be a huge benefit for Alzheimer’s patients.	1.00
Theme Unrelated to Food and Nutrition Popularization	Content unrelated to food, diet, nutrients and science popularization	Do sensory evaluators just keep tasting new foods?	1.00

**Table 2:** Distribution of content themes on the TikTok account of COFCO Nutrition Research Institute.

Content Themes	Frequency	Percent (%)
Practical Cooking Skills	10	12.35
Healthy Diet	33	40.74
Food Nutrition	14	17.28
Food and Environment	13	16.05
Nutrition Guidance for Targeted Groups	11	13.58
Total	81	100.00

**Table 3:** Descriptive distribution of user engagement metrics for coded videos.

Engagement Metrics	Count	Min.	Max.	SD.	Median
Number of Likes	81	50.00	7207.000	1423.941	726.000
Number of Shares	81	2.000	1940.000	236.991	42.000
Number of Comments	81	4.000	904.000	172.643	46.000
Number of Subscription Increases	81	0.000	3589.000	567.367	23.000

**Table 4:** Descriptive analysis of user engagement metrics on content themes.

Content Theme	Engagement Metrics	Min.	Max.	SD.	Median
Practical Cooking Skills	Number of Likes	77	7207	2508.037	1529.5
	Number of Shares	4	1940	603.248	80.5
	Number of Comments	4	800	237.765	58
	Number of Subscription Increases	4	3589	120.626	81
Healthy Diet	Number of Likes	50	4631	1106.952	561
	Number of Shares	3	211	67.784	45
	Number of Comments	7	687	126.811	34
	Number of Subscription Increases	1	1461	352.122	20
Food Nutrition	Number of Likes	59	3017	958.839	469.5
	Number of Shares	5	159	39.224	22
	Number of Comments	10	510	129.973	35
	Number of Subscription Increases	3	1143	301.99	16.5
Food and Environment	Number of Likes	105	5572	1549.103	929
	Number of Shares	2	541	150.671	54
	Number of Comments	12	904	249.23	114
	Number of Subscription Increases	1	2696	751.944	29
Nutrition Guidance for Targeted Groups	Number of Likes	59	2355	755.548	479
	Number of Shares	9	518	153.130	29
	Number of Comments	6	547	158.261	61
	Number of Subscription Increases	0	576	170.418	11

**Table 5:** Predicting engagements with jargon explanation, presenting conclusions and recommendations.

Variables	Model 1: Number of Subscription Increases		Model 2: Number of Shares		Model 3: Number of Comments	
	Regression Coefficient	OR Value	Regression Coefficient	OR Value	Regression Coefficient	OR Value
**Explanation of Jargon**						
Metaphor	−0.974 ** (−3.647)	0.378	--------			
Personification	0.441 * (2.262)	1.554	--------			
Science Visualization	0.478 ** (3.534)	1.613	--------			
Intercept	5.201	43.408	--------			
Likelihood Ratio	X^2^ (3) = 24.073 *p* = 0.000		--------			
McFadden R2	0.023		--------			
**Scientific Message Attributes**						
Conclusion Presence	--------		−0.582 * (−2.511)	0.559	0.44 (1.876)	1.544
Recommendation Presence	--------		0.190 (0.830)	1.209	0.924 ** (4.025)	2.518
Intercept	--------		5.097 (9.690)	163.540	2.459 (4.663)	11.698
Likelihood Ratio	--------		X^2^ (2) = 7.670 *p* = 0.022		X^2^ (2) = 19.341 *p* = 0.000	
McFadden R2	--------		0.008		0.021	

* *p* < 0.05, ** *p* < 0.01, z values in brackets, negative binominal regression.

**Table 6:** Negative binomial regression of content themes on engagement metrics.

Content Themes	Number of Likes	Number of Shares	Number of Comments	Number of Subscription Increases
Intercept	5.894 ** (58.150)	3.795 ** (37.227)	3.909 ** (38.405)	4.430 ** (43.581)
Practical Cooking Skills	1.866 ** (6.799) OR = 6.595	1.960 ** (7.051) OR = 7.098	1.064 ** (3.824) OR = 2.899	2.010 ** (7.239) OR = 7.463
Healthy Diet	0.959 ** (5.493) OR = 2.610	0.511 ** (2.909) OR = 1.667	0.398 * (2.267) OR = 1.489	0.772 ** (4.412) OR = 2.165
Food Nutrition	0.915 ** (3.801) OR = 2.497	−0.221 (−0.907) OR = 0.802	0.363 (1.498) OR = 1.437	0.414 (1.713) OR = 1.513
Food and Environment	1.357 ** (5.468) OR = 3.884	0.782 ** (3.138) OR = 2.187	1.311 ** (5.269) OR = 3.710	1.395 ** (5.611) OR = 4.033
Nutrition guidance for Targeted Group	0.777 ** (2.916) OR = 2.175	0.762 ** (2.848) OR = 2.143	0.772 ** (2.888) OR = 2.165	−0.161 (−0.600) OR = 0.851
Likelihood ratio McFadden R2	X^2^ (4) = 10.790, *p* = 0.029 0.008	X^2^ (4) = 33.267, *p* = 0.000 0.036	X^2^ (4) = 11.473, *p* = 0.022 0.013	X^2^ (4)= 32.755, *p* = 0.000 0.031

* *p* < 0.05, ** *p* < 0.01, z values in brackets, negative binominal regression.

## Data Availability

The datasets analyzed for this study would be available upon request with permission of the COFCO Research Institute.
